# Isolated Idiopathic Intra-articular Necrotizing Granuloma in a Native Knee Joint: A Rare Case Requiring a Diagnosis of Exclusion

**DOI:** 10.7759/cureus.109030

**Published:** 2026-05-17

**Authors:** Vanessa C Heck, Benjamin E Heck, Logan Van Poucke, Holly C Heck, Brian D Holmes, Alexander D Keith, Bruce E Heck

**Affiliations:** 1 College of Health and Human Services, University of Toledo, Toledo, USA; 2 College of Medicine and Life Sciences, University of Toledo, Toledo, USA; 3 College of Medicine, University of Illinois, Chicago, USA; 4 College of Natural Sciences and Mathematics, University of Toledo, Toledo, USA; 5 College of Medicine, Northeast Ohio Medical University, Rootstown, USA; 6 Department of Orthopedic Surgery, University of Toledo Medical Center, Toledo, USA; 7 Department of Orthopedics and Sports Medicine, Northwest Ohio Orthopedics and Sports Medicine, Findlay, USA

**Keywords:** diagnosis of exclusion, granuloma, idiopathic, knee, necrotizing granuloma, rare

## Abstract

Idiopathic necrotizing granuloma localized to a native knee joint is an exceedingly rare entity. While non-necrotizing granulomas as defined by organized epithelioid histiocytes and multinucleated giant cells without central necrosis are more commonly associated with autoimmune, foreign body, or inflammatory conditions, the presence of necrosis raises concern for more aggressive pathology, particularly infectious etiologies such as mycobacterial or fungal disease. Accordingly, idiopathic necrotizing granuloma remains a diagnosis of exclusion, requiring systematic evaluation to rule out infectious, post-procedural, autoimmune, and systemic granulomatous causes. This case report presents a 54-year-old man with a necrotizing granuloma within the suprapatellar pouch of the right knee noted at the time of arthroscopy for a medial meniscal tear and osteoarthritis. The patient did complain of pain directly in the area corresponding to the mass, the peripatellar region, as well as the medial joint line. He was initially treated with conservative measures including acetaminophen, non-steroidal anti-inflammatory, methylprednisolone, a knee sleeve, and physical therapy. After failure to improve, the patient requested to follow up with our practice due to increasing pain and difficulty with ambulation for surgical consideration. Radiographs and MRI revealed joint space narrowing consistent with osteoarthritis and a medial meniscal tear without mention of a mass. The patient requested surgical treatment secondary to pain with activities and difficulties with his vocation as a laborer. All physicians must be mindful that necrotizing granulomas indicate more aggressive tissue injury and should raise immediate concern for infection or other aggressive pathology mandating a comprehensive diagnostic evaluation to rule out systemic conditions and to prevent a delay in diagnosis or treatment. Knowledge that necrotizing granuloma can present as a painful mass within the knee without prior surgery, prior injection, infection, or systemic disease to our knowledge has not been described.

## Introduction

Idiopathic necrotizing granuloma localized to a native knee joint is an exceedingly rare entity. Granulomas are organized collections of epithelioid histiocytes and multinucleated giant cells that form in response to persistent inflammatory stimuli [1-3]. Non-necrotizing granulomas, characterized by the absence of central necrosis and preservation of surrounding cellular architecture, are more commonly encountered and are often associated with autoimmune conditions, foreign body reactions, and drug reactions and tend to be associated with non-infectious inflammatory conditions [2,3]. In contrast, necrotizing granulomas demonstrate central areas of acellular necrosis within an organized granulomatous framework [2]. The presence of necrosis raises concern for more aggressive pathology, most commonly infectious etiologies such as mycobacterial or fungal disease, but also vasculitis, severe immune-mediated processes, and rarely malignancy-associated granulomatous reactions [2,4,5]. A diagnosis of idiopathic necrotizing granuloma is therefore one of exclusion and requires a systematic evaluation to eliminate more common causes, including prior surgical or foreign body reaction, occult infection, post-injection inflammatory response, autoimmune or systemic inflammatory disease, and extrapulmonary granulomatous conditions [2,6-11].

This case report presents a rare instance of idiopathic necrotizing granuloma arising within the suprapatellar pouch of a native knee joint in a 54-year-old man, highlighting the importance of comprehensive diagnostic evaluation and thorough intraoperative assessment even when preoperative imaging does not reveal concerning findings.

## Case presentation

A 54-year-old male laborer sought care for increasingly progressive right knee pain following a slip while wearing flip flops in his garage five months previously. The patient did not fall and denied a history of knee pain prior to his twisting injury. He reported pain at rest and increased pain with activities of daily living, particularly during twisting or squatting while working as a laborer. The patient localized pain primarily to the anterior, superolateral aspect of the knee just proximal and lateral to the patella and to the medial aspect of the knee. The pain quality was described as aching.

He stated the pain was 9 on a scale from 0 to 10. When describing the pain, he stated it was constant. He reported waking up with the pain. He was taking over-the-counter non-steroidal anti-inflammatory with mild relief and tried a knee brace without relief. Physical modalities of ice, heat, and exercise did not improve the condition. His past medical history was unremarkable, and he denied a history of past knee pain. The patient's past surgical history was unremarkable for any knee surgery or injections to the right knee. He was on no medication and had no allergies. Family medical history was significant for osteoarthritis in his mother and maternal grandmother, but was otherwise unremarkable for any other joint diseases, inflammatory diseases, or autoimmune diseases. Socially, the patient reported occasional alcohol use and never smoked. Although he reported that his original symptoms started after slipping in his garage in flip flops but not falling at which time he thought he had strained his knee and it would get better, the patient stated that over the next five months, his knee pain progressively worsened. Due to ongoing pain, he sought care with a nurse practitioner and tried oral methylprednisolone but failed to show improvement or resolution of the problem. Over the next several weeks, the right knee became so painful that he could not work and the pain would awaken the patient from sleep. The patient then contacted the corresponding author for evaluation. 

**Figure 1:**
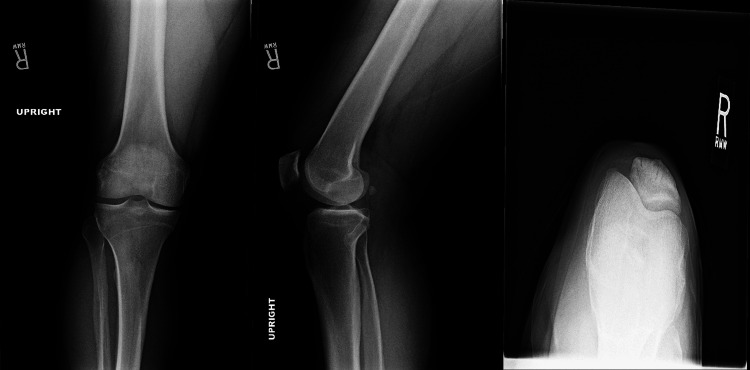
Anteroposterior, lateral, and sunrise views of the right knee demonstrating mild medial joint space narrowing on the anteroposterior view consistent with osteoarthritis noted at the time of surgery.

**Figure 2:**
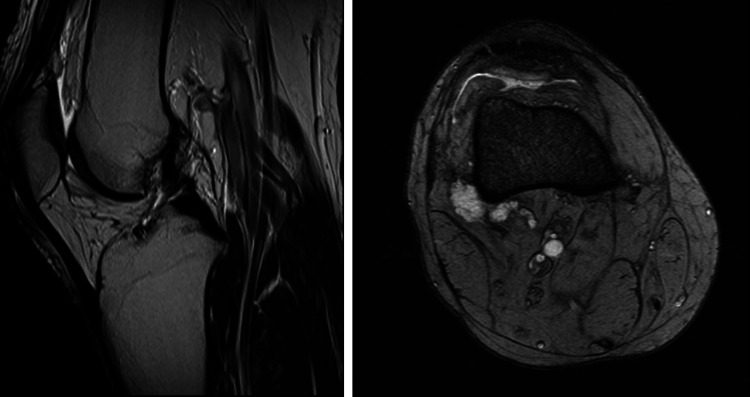
T2-weighted sagittal MRI of the right knee demonstrating the absence of a characteristic hyperintense necrotic center or surrounding inflammatory edema within the superolateral suprapatellar pouch corresponding to the necrotizing granuloma identified intraoperatively.

On physical examination, the patient was a 6’2”, 273-lb male laborer with a healthy appearance. The right lower extremity exam started at the hip which was normal to visual inspection and had no pain to palpation and no pain with a full range of motion. The right knee to inspection had no signs of redness, abnormal skin markings, or erythema. There appeared to be mild swelling with knee effusion. There was medial joint line tenderness to palpation with positive McMurray's exam as well as discomfort and crepitance with patellofemoral load testing. There were no palpable masses or other abnormalities identified in the superolateral aspect of the knee, where the patient also complained of pain, though there was pain to direct palpation in that area. The patient had no pain with passive extension of the toes or foot. He had no other pain, skin changes, or bony prominences on either foot or upper or lower extremities. All lower extremity compartments were soft. The patient had intact pulses and neurovascular function. Radiographs of the right knee revealed no foreign body, exostosis, or tumor, though they did demonstrate evidence of medial joint space narrowing with marginal osteophytes consistent with arthritis (Figure 1). An MRI obtained showed signs of chondromalacia with narrowing of the medial joint compartment and myxoid change of the medial meniscus with tear of the posterior horn at the junction with the body extending to the inferior articular surface with mild synovial thickening and effusion, but no report of an intra-articular mass and no report of increased signal in or around the area of the mass noted at the time of surgery (Figure 2). The patient had risks and benefits of continued conservative treatment including injections versus surgical treatment, and he requested surgical intervention due to his failure to improve over five months with mechanical symptoms and worsening condition.

**Figure 3:**
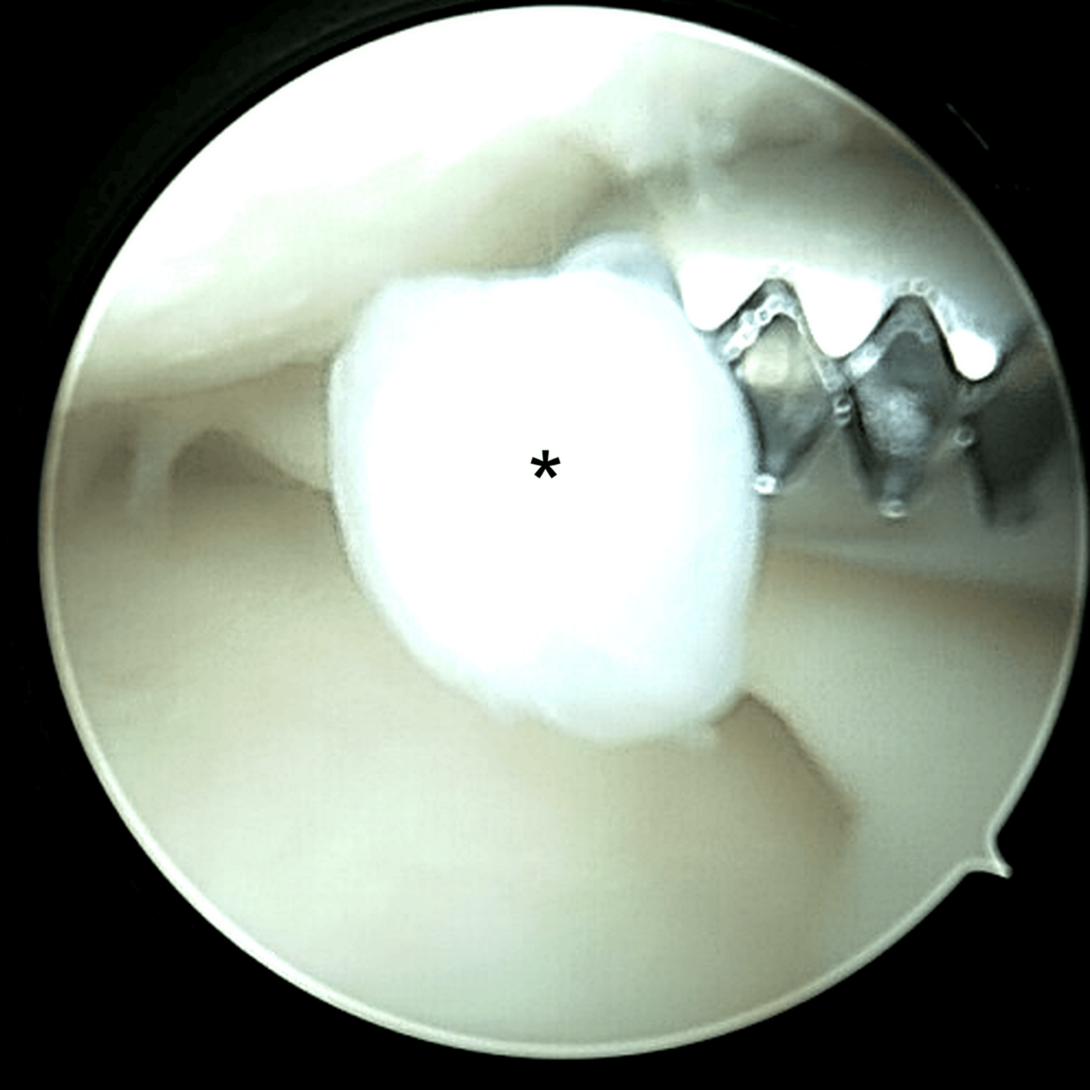
Arthroscopic image from the anterolateral portal demonstrating a 4 × 1 mm chondral loose body, labeled with asterisk, within the medial compartment of the right knee.

The patient was taken to surgery once medical clearance was obtained. Preoperatively, the patient received Ancef 2 gram intravenously. He had general laryngeal mask anesthesia in the supine position. Thromboembolic stockings and compression pumps were placed on the non-operative side. The patient had a tourniquet applied to the proximal right thigh, though it was never inflated. He was prepped and draped in sterile fashion. Time-out was performed with initials seen on the operative extremity. Respectively, anteromedial and anterolateral portal sites were injected with 10 cc of 0.5% Marcaine with epinephrine. Portal sites were developed, and the arthroscope was inserted in the anterolateral portal. Upon entering the joint, there was evidence of clear synovial fluid, synovial hypertrophy, and fibrillated chondromalacia of the patella femoral joint, more so along the lateral facet of the patella. The anterior cruciate ligament was intact. The medial compartment revealed evidence of a 4 × 1 mm chondral loose body (Figure 3) and complex medial meniscus tear and fibrillated chondromalacia of the medial femoral condyle in this compartment. The patient underwent partial medial meniscectomy and chondroplasty in a smooth fashion. The lateral compartment revealed degenerative fraying of the lateral meniscus and fibrillated chondromalacia of the lateral femoral condyle for which debridement was performed. Next attention was directed to the patellofemoral joint, where the patient underwent chondroplasty of the patella with special attention to the area of the patient's pain in the superolateral area of the suprapatellar pouch just superior and lateral to the patella.

**Figure 4:**
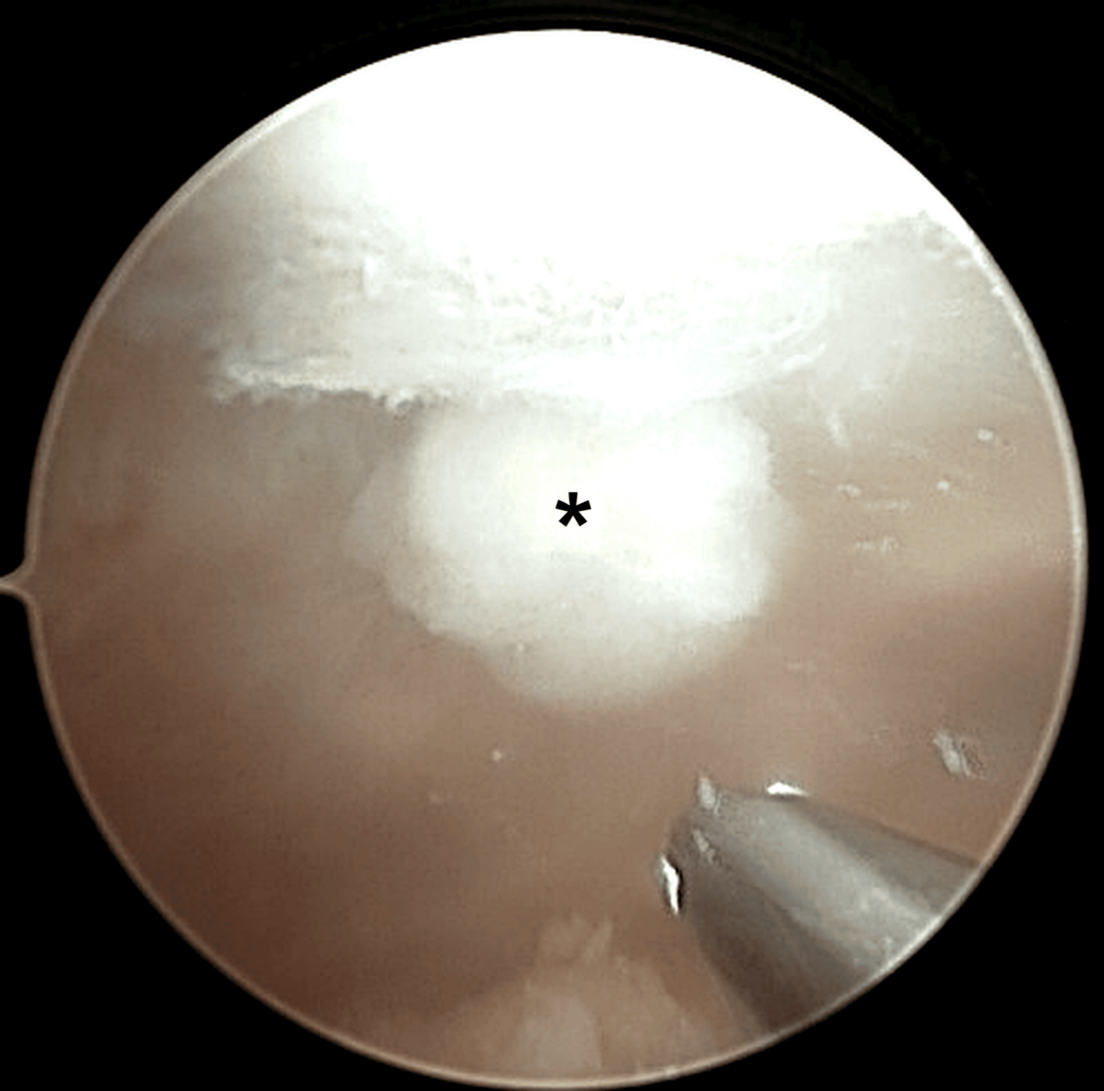
Arthroscopic image from the anterolateral portal of the right knee demonstrating firm necrotizing granuloma, labeled with asterisk, unresectable with shaver.

**Figure 5:**
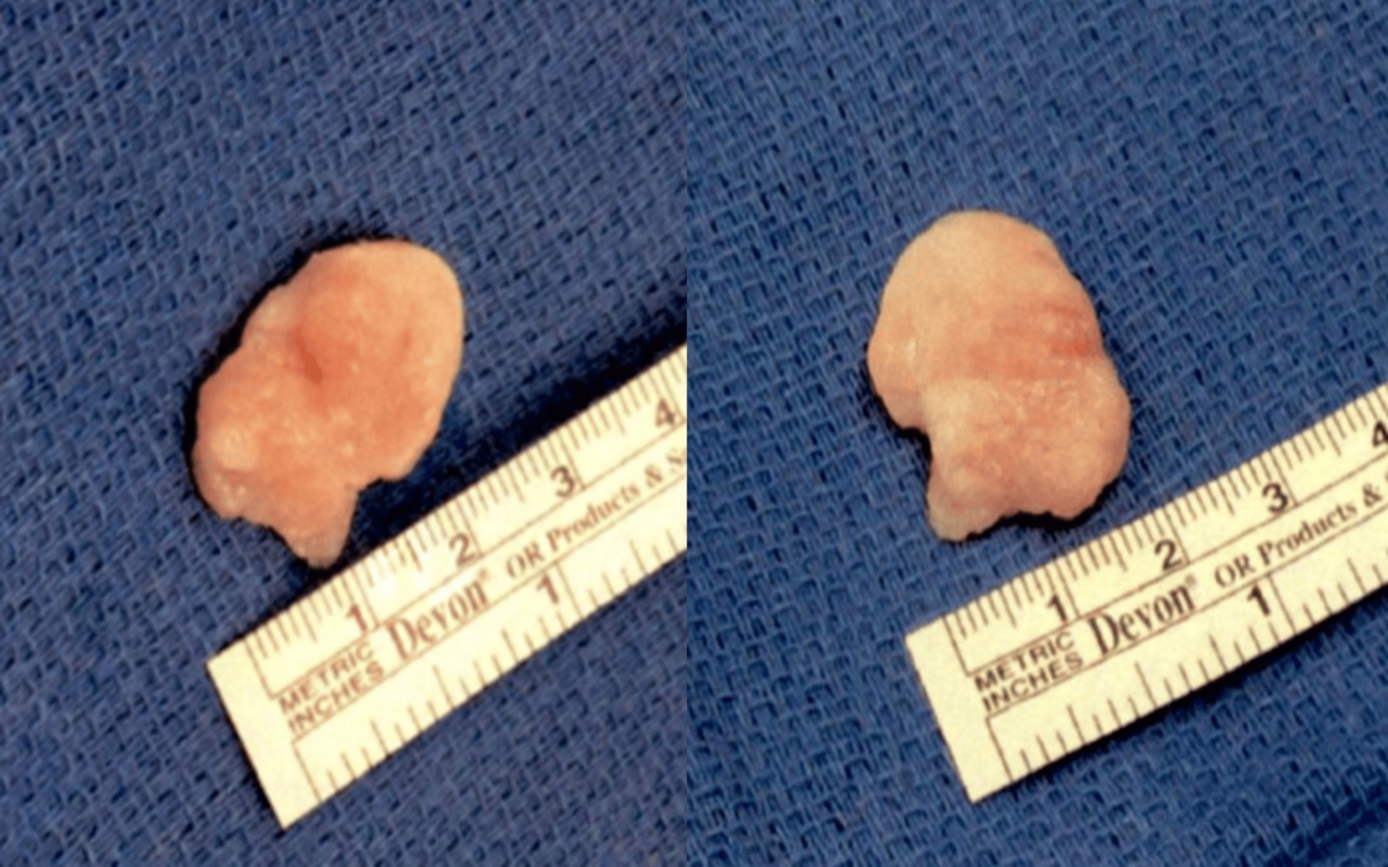
Intraoperative photographic image of an approximately 3-cm-long and 2-cm-wide tan-colored mass removed from the right knee identified histologically as necrotizing granuloma from two separate labs including the Mayo Clinic.

There was evidence of a mass approximately 3 cm in diameter. The mass was probed from multiple sides and appeared firmly attached to the capsule. Next, a synovial trap was inserted into the shaver suction line, and a 5.5 mm shaver was used to attempt excision; however, the tissue was too firm, and the shaver could not penetrate the mass for excision (Figure 4). Next, an arthroscopic scissor was inserted at the base of the mass, attached to the capsule, and an excision was performed. A large pituitary rongeur was used as a grasper to deliver the mass through an enlarged medial portal site. Multiple photographs were taken on the back table demonstrating an approximately 3 × 2 cm tan-colored mass (Figure 5). Both the mass and the synovial trap shavings were sent to pathology. The patient underwent extensive tricompartmental synovectomy with evaluation of all compartments and corners of the knee without other masses or abnormalities noted. Instrumentation was removed, portal sites were closed with nylon, and a sterile dressing was applied.

**Figure 6:**
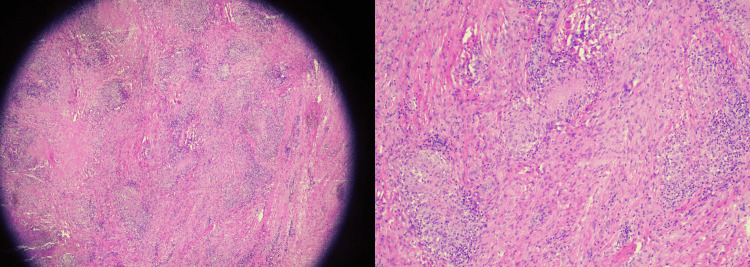
Histopathologic evaluation of the excised suprapatellar mass demonstrating necrotizing granulomatous inflammation within the dense fibrous tissue. Special stains including GMS, PASLG, Fite-Faraco, and AFB stains were negative for fungal organisms and mycobacteria. No evidence of malignancy was present. GMS: Grocott methenamine silver; PASLG: periodic acid-Schiff with light green; AFB: acid-fast bacillus

During the follow-up, the patient was already feeling better at two weeks postoperatively. There were no signs of infection. The pathologic specimen was reviewed from two separate laboratories including the Mayo Clinic with the same finding. Gross inspection revealed a 2.8 × 2.5 cm tan-white tissue. Histological sections showed a dense fibrous tissue with extensive necrotizing granulomatous inflammation with Grocott methenamine silver (GMS), periodic acid-Schiff with light green (PASLG), Fite-Faraco, and acid-fast bacillus stains showing no evidence of microorganisms, including mycobacteria or fungi (Figure 6). There was no evidence of malignancy. Due to concern for underlying autoimmune, systemic, and infectious disease, the patient had additional bloodwork and testing. Chest X-ray and blood work including complete blood count, metabolic panel, erythrocyte sedimentation rate, C-reactive protein, Lyme titer, anti-nuclear antibody, rheumatoid factor, and QuantiFERON-TB Gold Plus (QFT-Plus) were normal.

The patient resumed normal activity at six weeks post-op as a laborer for a city and had relief of pain in the superolateral aspect of the knee at the previous location of the mass. He will continue with follow-up to ensure no recurrence.

## Discussion

Periarticular masses demonstrating necrotizing granulomatous inflammation pose a unique diagnostic challenge [2,12]. Necrotizing granulomas are classically associated with mycobacterial and fungal infections [2,6,12,13]. They are reactive inflammatory lesions that do not themselves undergo malignant transformation; however, granulomatous reactions may be associated with underlying malignancies, may mask neoplastic cells, and may form lesions that mimic tumors on imaging [2,14]. A substantial proportion of cases remain culture-negative despite comprehensive evaluation and testing, and non-infectious inflammatory and iatrogenic etiologies must also be considered [6,13].

Rheumatoid nodules, sterile necrotizing granulomas characterized by central fibrinoid necrosis, occur in approximately 30-40% of patients with rheumatoid arthritis, particularly those with positive anti-cyclic citrullinated peptide (anti-CCP) antibodies or high rheumatoid factor titers [2,15-17]. Granulomatosis with polyangiitis (GPA), an antineutrophil cytoplasmic antibody (ANCA)-associated vasculitis, and sarcoid granulomatosis warrant additional consideration, as both can manifest with necrotizing granulomatous inflammation affecting tissues [5,11,14,18-20]. Additionally, hyaluronate-related granulomatous synovitis following viscous supplementation is a rare but recognized iatrogenic etiology [9,10,21]. Our patient had no underlying history of autoimmune disease, systemic disease, penetrating trauma, or treatment to his knee to cause an underlying necrotizing granuloma and, more importantly, was extensively worked up after the pathologic diagnosis to rule out an etiological factor without success.

Unfortunately, our patient had a delay in seeking medical care following a slip-and-twist injury to the knee, initially resulting in pain that was likely attributable to the mechanical injury itself. However, the persistence and progression of symptoms beyond the expected recovery period, in conjunction with the identification of a 3 cm mass, raised concern for an underlying pathologic process distinct from the original injury. While conditions such as arthritis aggravation and meniscal tear are two of the most common reasons for continued pain and problems following a twisting injury to the knee [22], other reasons for pain including fracture, ligament and tendon injury, referred pain from the hip or back, masses, and tumors must be distinguished either clinically, radiographically, or with advanced imaging [23,24]. This case presents a rare and to our knowledge first report of an idiopathic necrotizing granuloma presenting in the same knee after a twisting injury five months earlier. Most necrotizing granulomas of the musculoskeletal system present as painful masses, especially when involving nerves [25] or the knee joint [12] as occurred in our case. Although it is well documented in the literature that necrotizing granulomas secondary to infection of the musculoskeletal system can present with symptoms of pain [12,25], this is the first case report of a necrotizing granuloma of the knee without infection identified or other underlying systemic disease causing pain. 

Our patient had an idiopathic necrotizing granuloma without an identifiable underlying etiology despite aggressive clinical, serologic, microbiologic, and histologic workup. Fortunately, his pain localized to the area of the mass resolved after excision and has not recurred, which is similar to the experience of other authors who have noted that necrotizing granulomas that do not have a definitive etiology appear not to require any additional treatment and experience a favorable outcome [4]. This case highlights the importance of systemic evaluation to exclude common etiologies for necrotizing granuloma, including prior surgical or foreign body reaction, occult infection, post-injection inflammatory responses, autoimmune or systemic inflammatory disease, and rare extrapulmonary granulomatous conditions. Failure to perform a thorough exclusionary workup risks a missed diagnosis and improper treatment, which may result in further pain, functional impairment, and delayed recognition of more serious underlying pathology including malignancy. Also, the report highlights the importance of thoroughly evaluating the knee at the time of knee arthroscopy despite a report that reveals no mass and more vitally alerts all physicians to be mindful that necrotizing granulomas indicate more aggressive tissue injury and should raise immediate concern for infection or other aggressive pathology mandating a comprehensive diagnostic evaluation to prevent a delay in diagnosis or treatment. With negative radiograph and MRI and reports of such a mass, the arthritis and meniscal tear noted at the time of surgery could have resulted in the mass being easily overlooked which could lead to continued pain, dysfunction, and maltreatment. Interestingly, it has been concluded that degenerative joint lesions may be the cause of the reactive granulomatous form, but not the necrotizing granuloma form [26].

This report has several limitations. Despite an extensive diagnostic workup, the possibility of an occult infectious or systemic inflammatory process cannot be completely excluded, as some indolent etiologies may evade detection with currently available testing. Additionally, as a single case report, causal relationships cannot be definitively established, and the coexistence of degenerative arthritis changes and cartilage loose bodies as noted at the time of surgery (Figure 3) may represent either an incidental finding or an unrecognized contributing factor to the necrotizing granuloma. The rarity of isolated articular loose bodies causing necrotizing granulomas limits comparison with prior literature and restricts the ability to generalize these findings. Further accumulation of similar cases will be necessary to better characterize the pathogenesis, clinical behavior, and optimal management of this entity.

## Conclusions

This case highlights the importance of maintaining clinical suspicion for necrotizing granulomatous pathology in patients presenting with persistent knee pain, even in the absence of a palpable mass or definitive findings on advanced imaging. This exceptionally rare entity should be regarded as a diagnosis of exclusion, requiring systematic evaluation to eliminate infectious, autoimmune, post-procedural, and other secondary causes. Recognition that necrotizing granuloma may occur without overt signs of infection or systemic disease highlights the need for thorough diagnostic assessment and continued clinical surveillance. Increased awareness of this presentation may facilitate earlier identification, appropriate management, and improved patient outcomes in similarly atypical cases.
